# How Government Grinds the Poor

**Published:** 1913-01-25

**Authors:** 


### HOW GOVERNMENT GRINDS THE POOR.

Englishmen have a world-wide reputation as administrators. Up to thirty years ago no Government could have attempted grandmotherly legislation and lived in this country. To-day the people appear to have grown so soft that self-reliance and pride of personal effort are, we fear, dying forces, when nearly everybody will take with both hands doles or other destructors of personal independence from the Government. So the English nation, unless it speedily wakes up, will find itself in the steel arms of Government control.

However unpleasant the lesson may prove in practice, first experiences of the National Insurance Act seem calculated to convince the workers throughout the country that the first result of Government machinery in action is to grind the poor. The inquest held upon the workman who, though insured, died of strangulated hernia because he and his friends relied upon the Insurance Act's machinery to bring him adequate medical aid, is only typical of many cruelties perpetrated on the poor by the machinery of the Poor Law. How many ratepayers realise that similar cases to that of the insured man just referred to are constantly happening? One such case came to our notice a fortnight ago, where death would have resulted but for the personal service freely rendered by a friendly surgeon. The delays in the admission of patients to the infirmaries throughout London are frequently dangerous to the life of the poor. The action of the relieving officers is sometimes brutal beyond belief. They afflict the poor by refusing them relief so long as they exhibit self-respect. The cruelties and moral injury done to the poorer residents of London alone by the Government machine in Poor-Law matters is enough to make angels weep. We shall shortly attempt to expose the abominable system in all its working ugliness. Is the report of the Poor-Law Commission forgotten, and can the present Ministry, and Mr. John Burns especially, venture to go to the country without making an attempt to remedy the notorious abuses of the system? How long will the ratepayers of the Metropolis be content to suffer these things and to pay for the continuance of this grinding of the poor?

